# Outcome of hyaluronic acid gel injection in glans penis for treatment of lifelong premature ejaculation: A pilot study

**DOI:** 10.1080/2090598X.2022.2100580

**Published:** 2022-07-19

**Authors:** Ahmed Sakr, Hazem Elgalaly, Mohamed M. Seleem, Mostafa Kamel, Ahmed I. El-Sakka, Ibrahim M. Ibrahim

**Affiliations:** a Faculty of Medicine, Urology Department, Zagazig University, Zagazig, Egypt; b Faculty of Medicine, Urology Department, Suez Canal University, Ismailia, Egypt

**Keywords:** Glans penis injection, premature ejaculation, hyaluronic acid

## Abstract

**Objective:**

To assess safety and efficacy of hyaluronic acid (HA) gel injection in glans penis for treatment of premature ejaculation (PE) using our new five puncture technique.

**Patients and methods:**

This is a prospective, non-randomized clinical trial on HA gel injection in glans penis for all patients with lifelong PE; all patients were circumcised having heterosexual normal marital life and sexually active. Patients with history of ejaculatory medication use within the previous 3 months, psychiatric disorders, erectile dysfunction, lower urinary tract symptoms (LUTS) due to prostatitis and acquired PE were excluded from the study. A local anesthetic was applied to the skin of glans penis for 30 minutes before the injection of 2 ml HA in glans penis via 30-gauge needle using our new Five-puncture technique. Intra-vaginal ejaculatory latency time (IELT) was measured at 1, 3, 6 and 12 months after injection.

**Results:**

Thirty patients completed our study follow up schedule. Mean age of the patients was 41.72 ± 8.50, while mean age of female partner was 37.23 ± 8.54 years. IELT was highly significantly increased (*P*-value < 0.001) after HA gel injection from baseline, which was in maximum 37.83 ± 11.01 sec at baseline to 323.03 ± 42.06, 281.07 ± 41.05, 241.03 ± 43.09 and 235.6 ± 41.87 sec after 1, 3, 6 and 12 months, respectively, after injection. Three patients complained from discomfort at the site of injection, two from bullae formation at the site of injection and one from ecchymosis, and all resolved spontaneously after 1 week to 10 days after injection.

**Conclusion:**

HA gel injection in glans penis using our new five-puncture technique is a safe and effective method that ensures a modest long-term significant increase in IELT and improves ejaculatory control.

## Introduction

In men, premature ejaculation (PE) affects 20% of the population and is the most commonly self-reported sexually dysfunctional behavior among men [1].

American Urological Association Guidelines 2020 (AUA) recently introduced a new definition, defining PE as poor ejaculatory control and related discomfort as well as ejaculation within approximately 2 min after the start of penetrative sex [2].

PE can be classified into two categories: 1. Lifelong PE is when ejaculation occurrs within 30–60 seconds after beginning penetrative intercourse in most cases [3]. 5- Hydroxytryptamine 1A (5-HT1A) hypersensitivity and peripheral penile hypersensitivity may be the cause of lifelong PE [4]. 2. Acquired PE is when the ejaculatory latency is markedly reduced after previous normal sexual intercourse [3].

For the time being, the only short-acting selective serotonin reuptake inhibitor approved for treating PE is Dapoxetine. A combination of local anesthetics and antidepressants also gives good results [5].

Recurrence of PE after drug cessation and systemic side effects are the main drawbacks of these treatments [6].

In both Europe and the United States, hyaluronic acid derivatives are the most commonly used biodegradable fillers [7]. As a result, hyaluronic acid (HA) gel injections in glans penis have been developed and are currently being used to treat PE and lower the strength of the stimuli reaching sensory receptors in the penile skin [8].

Researchers found that HA injections increased intra-vaginal latency time (IELT) by up to 4.46 times in several tests and that this increase lasted for a long time (up to 5 years) [9,10]. Except for temporary discoloration and swelling of the glans, which returned to normal in 2 weeks, there were no other major side effects observed [10]. Paresthesia and hypoesthesia after glans penis augmentation (GPA) using HA filler are rare, and no cases of erectile dysfunction were encountered [11]. Severe complications such as glans necrosis due to vascular compromise after indirect glans augmentation via implantation of dermofat grafts or scaffolds between corpus spongiosum and the distal tip of corpus cavernosum [12] were not encountered with HA gel injection just in the deep dermis without any surgical dissection in glans penis for GPA.

The aim of the current study is to assess safety and efficacy of HA gel injection in glans penis using our new five-puncture technique to ensure the maximum equal distribution of the HA with least number of injections for treatment of PE.

## Patients and methods

After local ethics committee approval and informed consent from all patients were obtained, we conducted this prospective, non-randomized clinical trial between June 2020 and November 2021 on all patients with PE who met our inclusion criteria.

Sample size was calculated by assuming that the mean rated self-satisfaction was 90 ± 72 sec vs 372 ± 240 sec before vs after intervention, at 80% power and 95% CL. After adding 10% of possible drop out or loss during follow-up, the estimated sample size will be at least 34 cases, open epi.

Patients with lifelong PE were included in the study with, all patients were circumcised having heterosexual normal marital life and sexually active. Long-term PE was diagnosed based on standards from the International Society for Sexual Medicine (ISSM), using IELT stopwatch testing. Pre-assessment in the baseline period of 4 weeks, all individuals were required to have at least four sexual intercourses.

Exclusion criteria included 1. patients with history of ejaculatory medication use within the previous 3 months, 2. psychiatric disorders like schizophrenia, bipolar disorder and other psychoses, as well as addiction, 3. erectile dysfunction, 4. patients with lower urinary tract symptoms (LUTS) due to prostatitis, and 5. patients with acquired PE. A thorough history was taken about demographic data of the patients, sexual life and special habits.

### *Our new five-puncture technique of injection* (Figure 1)

**Figure 1.**
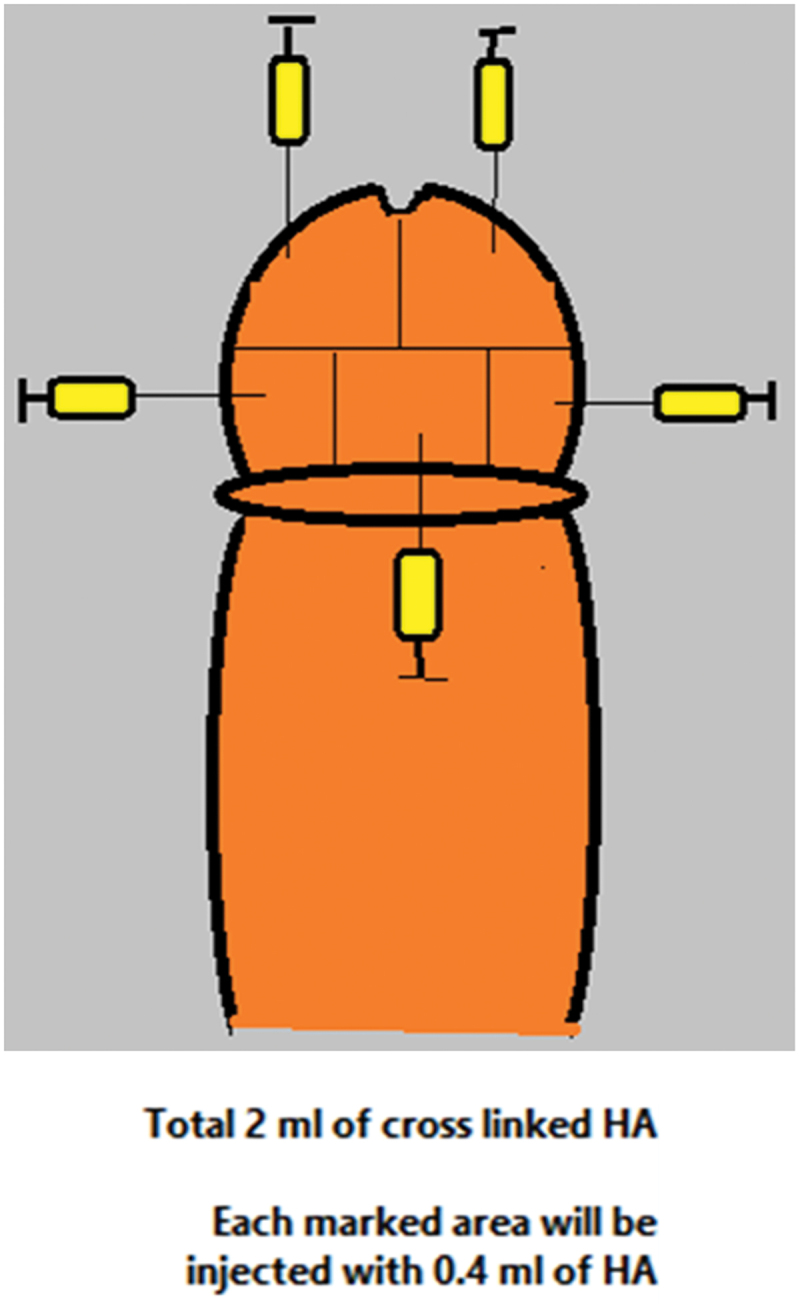
Our new five-puncture technique.

Xylocaine Jelly 2%:lidocaine 20 mg (Aspen, Sweden) was applied to the skin as a local anesthetic for 30 minutes before the injection of 2 ml hyaluronic acid (HA; STYLAGE® IPN Like TECHNOLOGY, VIVACY Laboratories, Paris, France) in glans penis via 30-gauge needle using our new five-puncture technique. The glans penis was divided by a horizontal line into two halves; the distal half was divided by a vertical imaginary line into two compartments, and the proximal half was divided into three compartments by two vertical imaginary lines, the above mentioned design was used to ensure the maximum equal distribution of the HA with least number of injections; each compartment was injected in the deep dermis with 0.4 ml HA with total 2 ml over the glans.

All patients’ IELTs were measured before and after the injection at 1, 3, 6 and 12 months. Our primary objective was safety and efficacy of HA gel injection in glans penis for treatment of PE, while the secondary objective was the advantages of the five-puncture technique over the previously published techniques.

### Statistical analysis

Data were analyzed using IBM SPSS 23.0 for windows (SPSS Inc., Chicago, IL, USA) and NCSS 11 for windows (NCSS LCC., Kaysville, UT, USA). Quantitative data were expressed as mean ± standard deviation (SD). Qualitative data were expressed as frequency and percentage. The following tests were done:

- Repeated measures ANOVA test was used when comparing repeated measures taken of more than two readings of normally distributed data, whereas not normally distributed data were compared using Friedman’s test.
- Wilcoxon signed-rank test was used for comparing the change in means from baseline of not normally distributed data.

Probability (P-value): P-value ≤ 0.05 was considered significant, P-value ≤ 0.001 was considered as highly significant and P-value > 0.05 was considered insignificant.

## Results

**Figure 2.**
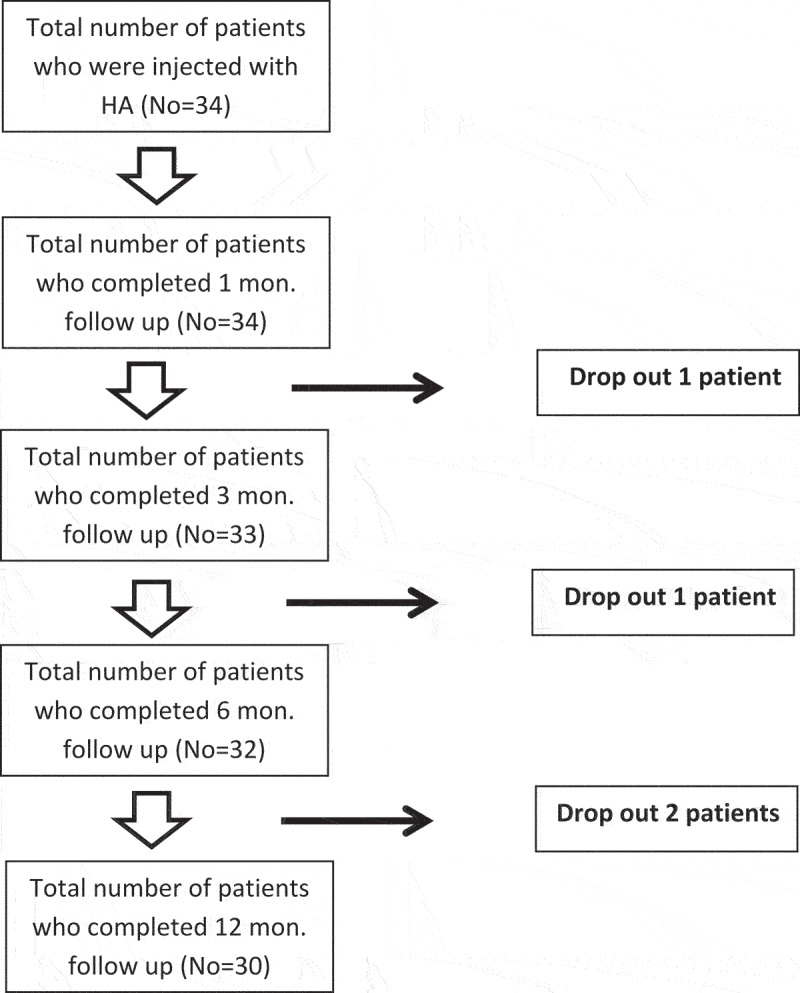
Flowchart of the patients.

A total of 34 patients with PE who met our inclusion criteria were included in the study, while only 30 patients completed the follow-up schedule (Figure 2).

Mean age of the patients in our study was 41.72 ± 8.50 (20 to 55 years), while mean age of female partners was 37.23 ± 8.54 (20 to 53 years). Average mean body mass index was 20.22 ± 3.89 kg/m^2^ with the other demographic data of the patients (Table 1).

**Table 1.** Demographic data of the patients.

Parameters	Mean ± SD	Range
Age of the patients in years	41.72 ± 8.50	20–55
BMI (Body mass index) kg/m^2^	23.22 ± 3.89	19–30
Age of the wife in years	37.23 ± 8.54	(20–53)
Duration of marriage in years	12.3 ± 4.31	(3–20)
Intercourse frequency/week	1.86 ± 0.76	1–3
IELT (sec)	37.83 ± 11.006	20–60

IELT, Intra-vaginal ejaculation latency time

IELT was highly significantly increased (*P*-value < 0.001) after HA gel injection from baseline, which was in maximum 60 sec at baseline to 378, 318, 306 and 300 sec after 1, 3, 6 and 12 months, respectively, after injection (Table 2).

**Table 2.** IELT (sec): baseline versus 1, 3, 6 and 12 months after injection.

	IELT baseline	IELT after 1 month	IELT after 3 month	IELT after 6 months	IELT after 12 months
Mean ± SD	37.83 ± 11.01	323.03 ± 42.06	281.07 ± 41.05	241.03 ± 43.09	235.6 ± 41.87
Range	20–60	240–378	180–318	180–306	175–300
P-value with baselineIELT		<0.001	<0.001	<0.001	<0.001

The percentage of patient’s satisfaction was 83.3% (25/30) at 6 and 12 months without significant difference over time. In responding partners, percentage of satisfaction was 70% (21/30) at 6 months and 66.7% (20/30) at 12 months, without significant difference over time.

Three patients complained from discomfort at the site of injection, two patients complained from bullae formation at the site of injection and one patient complained of ecchymosis; all of the previously mentioned complications were well tolerated by the patients and resolved spontaneously after 1 week to 10 days from day of injection (Table 3).

**Table 3.** Complications of injection.

		Patients	
		N	%
Discomfort at site of injection	No	27	90
	Yes	3	10
Bullae formation at site of injection	No	28	93.34
	Yes	2	6.66
Ecchymosis	No	29	96.66
	Yes	1	3.34

## Discussion

In spite of the fact that PE is still poorly understood, the primary pathophysiology is a highly complex psychological one. Using filler to enhance the glans penis might have the effect of decreasing the sensation of the glans penis by creating a barrier between stimuli and the receptor, as well as increasing self-esteem [13]. Using injectable soft tissue substitutes instead of surgery can be an economical and non-invasive way to fix contour flaws and add volume to the soft tissues around the body. Fillers for soft tissue augmentation should be non-antigenic, non-inflammatory, biocompatible, non-pyrogenic, easy to use, stable after injection, long-lasting but absorbable, non-migratory, natural-looking and not prohibitively expensive [14]. HA is a naturally occurring polysaccharide; in the dermal intercellular matrix of every species’ skin with the same chemical and molecular structure exists throughout the animal kingdom, as a result, using animal sources in humans without causing foreign body reactions is extremely biocompatible [15].

This study shows that the injection of HA gel in glans penis is safe and effective in the treatment of lifelong PE, after injecting 30 patients with 2 ml of HA, using the five-puncture technique to ensure the best distribution of HA and better surface area coverage with least possible puncture sites, leading to a remarkable and statistically significant increase in IELT.

This result can be attributed to a lowered glans penis sensory threshold [16]. The delayed digestion of this gel demonstrates that cross-linkage stabilization of the material can enhance the lifespan of the natural polymer by several hundred times without biocompatibility reduction. There is deterioration of the implant, but it exhibits an isovolemic degradation feature. As a result of the isovolemic degradation, a less concentrated hyaluronan network has a greater capacity to bind water, which permits the correction to be maintained even at low concentrations of the materials [17].

In our study, 1-, 3-, 6- and 12-month post-injection IELTs were statistically highly significantly increased compared with pre-injection baseline one. The mean IELT increased significantly from 37.83 ± 11.01 to 323.03 ± 42.06 sec after 1 month of injection and then dropped to 281.07 ± 41.05 sec after 3 months, then 241.03 ± 43.09 sec after 6 months and 235.6 ± 41.87 sec after 12 months but still remaining significantly higher than the baseline values (*P* < 0.001).

Compared to our current study, the previous studies reported a comparable data results in which Abdallah et al. [18] injected 60 men with PE, leading to an increase in the mean IELT from 127.2 ± 69.6 to 462.6 ± 471.6 sec after 1 month of injection and then dropped to 319.2 ± 211.2 sec. More comparable findings were documented by Kim et al. [13], showing a magnificent increase in the mean IELT from 96 to 330 sec after 6 months of injection; moreover, in 2016, two more studies were presented in the matter: one study was done by Mohee and Eardley [5] and the other was presented by Wein A et al. [16]; both of these studies agree on the remarkable increase in IELT after HA injection. If compared to the baseline pre-injection, it should be noted that Wein et al. [16] provided 5-year follow-up results, indicating a 2.43- to 4.46-folds rise in the IELT following injection. Again Littara et al. [19] noted an increase in the IELT with 3.32 times at 6 month post injection. Alahwany et al.’s [20] study found that, after receiving HA injections for a month, the IELT scores of 20 patients (67%) improved from their pre-treatment level, while the remaining trial participants (n = 10, 33%) did not have an improvement in IELT after a month of follow-up. Re-evaluating those subgroup of patients after 3, 6 and 9 months showed that IELT decreased steadily but still significantly higher than the baseline one [20].

Eventually, all of the previous studies agreed that there is significant increase in the IELT post-injection.

In fact, these results appears promising when compared to that of Dapoxetine 30 or 60 mg taken 1 to 2 hours before intercourse, which leads in a 2.5- and 3.0-fold increase in IELT, increased ejaculatory control, decreased discomfort and increased satisfaction [16].

There are no systemic side effects from HA injection, and there are no negative effects on sexual desire or fertility like there are with other medical treatments. Additionally, it has a beneficial impact on self-esteem and confidence in PE because of the larger glans [21].

The injection technique, on the other hand, was diverse, in which Kim and colleagues [13] developed the Fan shape technique that in contrast to Five-puncture technique may cause unequal distribution of HA which led in some cases to sudden glandular swelling and discoloration that resolved spontaneously within 2 weeks of injection. Two more techniques were described: one by Abdallah et al. [18] who developed the multiple puncture technique and the other by Littara et al. [19] who developed the three circle technique, both of which like our technique insure the best equal distribution of HA with better surface area coverage of injected matter but with more puncture sites that carry risk of glandular bruising which was documented in 7 cases in the study carried by Abdallah et al. [18].

The two-circle technique developed by Alahwany et al. [20] in which authors targeted at one circle the proximal half of dorsal surface of the penis at three puncture sites, while the other circle targeted the ventral surface of the glans at the frenulum in two more puncture sites to ensure the best equal distribution of HA with better surface area coverage of the glans like our Five-puncture technique but with the risk of urethral or frenular artery injury.

In our study, three patients complained from discomfort at the site of injection, two patient complained from bullae formation at the site of injection and one patient complained of ecchymosis, all of the previously mentioned side effects were well tolerated by the patients and resolved spontaneously after 1 week to 10 days from the day of injection. Abdallah et al. [18] reported in 30% of the cases with adverse reactions of ecchymosis, bruise at the site of injection and swelling with sudden discoloration of glans that resolved within 2 weeks spontaneously. Alahwany et al. [20] documented that 6 out of 30 cases showed similar adverse effect as in our study.

Limitations of our study included short follow-up period and being a cohort not a randomized controlled trials (RCTs) lacking a control arm. Further studies with longer follow-up in RCTs are definitely needed for more consistent results.

## Conclusion

Glans penis injection with HA gel using our new Five-puncture technique is a safe and effective method that ensures a modest long-term significant increase in IELT and improves ejaculatory control.
